# Behavior Change Training for Health Professionals: Evaluation of a 2-Hour Workshop

**DOI:** 10.2196/42010

**Published:** 2022-11-18

**Authors:** Barbara Mullan, Caitlin Liddelow, Darren Haywood, Hayley Breare

**Affiliations:** 1 enAble Institute Faculty of Health Sciences Curtin University Bentley Australia; 2 Western Australian Cancer Prevention Research Unit Curtin University Bentley Australia; 3 School of Population Health Curtin University Bentley Australia; 4 School of Psychology University of Wollongong Wollongong Australia; 5 Department of Mental Health St Vincent's Hospital Melbourne Fitzroy Australia

**Keywords:** behaviour change, psychology, psychological, BCT, health professional, health care professional, medical education, health care provider, continuing education, professional development, theory of planned behaviour, COM-B, workshop, intervention, clinical practice

## Abstract

**Background:**

Rates of noncommunicable diseases continue to rise worldwide. Many of these diseases are a result of engaging in risk behaviors. Without lifestyle and behavioral intervention, noncommunicable diseases can worsen and develop into more debilitating diseases. Behavioral interventions are an effective strategy to reduce the burden of disease. Behavior change techniques can be described as the “active ingredients” in behavior change and address the components that need to be altered in order for the target behavior to change. Health professionals, such as pharmacists and nurses, can engage in opportunistic behavior change with their patients, to encourage positive health behaviors.

**Objective:**

We aimed to develop, implement, and evaluate a behavior change workshop targeted at health professionals in Australia, with the goal of increasing knowledge of behavior change techniques and psychological variables.

**Methods:**

A prospective study design was used to develop and evaluate a 2-hour behavior change workshop targeted at health professionals. The workshop was developed based on the Capability, Opportunity, Motivation, and Behavior Model and had five core objectives: (1) to detail the role of health professionals in delivering optimal care, (2) to demonstrate opportunities to change behavior, (3) to describe principles of behavior change, (4) to explain behavior change techniques, and (5) to determine the most appropriate behavior change techniques to use and when to use them. A total of 10 workshops were conducted. To evaluate the workshops and identify any potential long-term changes in behavior, we collected pre- and postworkshop data on knowledge and psychological constructs from the attendees.

**Results:**

A final sample of 41 health professionals comprising general practitioners, nurses, and pharmacists completed the pre- and postworkshop surveys. Following the workshops, there were significant improvements in knowledge of behavior change techniques (*t*_40_=–5.27, *P*<.001), subjective norms (*t*_40_=–3.49, *P*=.001), descriptive norms (*t*_40_=–3.65, *P*<.001), perceived behavioral control (*t*_40_=–3.30, *P*=.002), and intention (*t*_36_=–3.32, *P*=.002); each had a large effect size. There was no significant difference in postworkshop attitude (*t*_40_=0.78, *P*=.44). The participants also found the workshops to be highly acceptable.

**Conclusions:**

A 2-hour, theoretically informed workshop designed to facilitate the use of behavior change techniques by health professionals was shown to be largely effective. The workshops resulted in increases in knowledge, descriptive and subjective norms, perceived behavioral control, and intention, but not in attitude. The intervention was also shown to be highly acceptable, with the large majority of participants deeming the intervention to be needed, useful, appropriate, and applicable, as well as interesting and worth their time. Future research should examine the lasting impacts of the workshop on health professionals’ practices.

## Introduction

Rates of noncommunicable diseases such as cancer, coronary heart disease, and diabetes continue to rise worldwide and account for 71% of deaths globally [1]. Many of these diseases are a result of engaging in unhealthy behaviors such as smoking, excessive alcohol consumption, and being sedentary. Cancer is one of many major chronic diseases that impacts individuals, families, communities, and economies. In Australia, 42% of the cancer burden is attributable to personal attributes and engagement with risk behaviors [2]. In 2021, it was estimated that more than 1 million individuals were impacted by either living with or having lived with cancer in Australia alone. Without lifestyle and behavioral intervention, noncommunicable diseases can worsen and develop into more debilitating diseases. For many chronic diseases, using behavioral interventions can be an effective strategy for reducing the burden of disease in Australia [3,4].

There are a range of theoretical frameworks that have been used to understand and predict behavior and inform the development of behavioral interventions. One popular framework is the Capability, Opportunity, Motivation, and Behavior (COM-B) model [5]. The COM-B model provides a framework to inform the choice and use of behavioral interventions. The COM-B model proposes that 3 components are integral in the production, and thereby changing, of behavior: capability (ie, a person’s perception of whether they are physically and psychologically able to complete the behavior), opportunity (ie, the physical and social opportunity to complete the behavior), and motivation (ie, the desire or need to complete the behavior over competing behaviors) [5]. A range of interventions have successfully used behavior change approaches targeted at improving capability, opportunity, or motivation with the goal of changing behavior [6]. The application of behavior change approaches can be implemented at the population level, through large-scale interventions, or on the individual level, through opportunistic implementation.

Recently, there have been calls for health professionals, such as pharmacists and nurses, to engage in opportunistic behavior change [7]. Public health policies from the National Health Service of the United Kingdom, such as Make Every Contact Count, promote the use of behavior change strategies and interventions by health professionals to engage with patients who may need additional assistance managing their health [8]. Keyworth et al [7] found that in practice, health professionals did not deliver opportunistic behavior change interventions on 50% of occasions when they were perceived as necessary. Health professionals reported a number of barriers to delivery of these interventions, such as beliefs about their capability and the consequences of these interactions; views on their professional role and identity; and more discipline-specific barriers, such as prioritization, time pressures, and workload pressures [9].

Among approaches that have a strong evidence base for successfully promoting healthy behaviors among patients and changing their behavior [10,11] are behavior change techniques [12]. Behavior change techniques can be described as the “active ingredients” in behavior change and often focus on the components that need to be altered in order for the target behavior to change. Behavior change techniques have been successfully used in previous interventions in both patients and health professionals [13]. A 2009 meta-analysis showed that interventions aimed at patients were more effective in improving medication adherence when active behavior change techniques were used [14]. Similarly, health care provider–led interventions that incorporated behavior change techniques, such as educating patients on the consequences of nonadherence, were successful in increasing the odds of medication adherence in patients with acute coronary syndrome by 54% [15]. Finally, a systematic review of reviews showed that behavior change interventions aimed at changing the practice behavior of health professionals were successful when they were interactive and multifaceted [16].

The aim of this study was to develop, implement, and evaluate a 2-hour behavior change workshop based on the COM-B model, targeted at health professionals in Western Australia. More specifically, the workshop was targeted at health professionals who treat or encounter patients experiencing various chronic diseases. The Theory of Planned Behavior variables [17] was used to obtain a deeper understanding of health professionals’ attitudes (ie, how positively or negatively they view engaging in a behavior), subjective norms (ie, how likely it is that others similar to them engage or believe they should engage in the behavior), perceived behavioral control (ie, how much control they have over engaging in the behavior), and intention (ie, whether they intend to change their behavior) regarding behavior change techniques and using them in practice. This theory is widely used in behavior research and suggests that the combination of attitudes, subjective norms, and perceived behavioral control predicts the intention to engage in a behavior. Intention and perceived behavioral control are then said to significantly predict engagement in the target behavior [17]. Through the piloting of the workshops, we hypothesized that health professionals would report a pre- to postworkshop increase in knowledge of behavior change techniques and the Theory of Planned Behavior variables. These variables are commonly used to assess and inform the likelihood of people engaging in target behaviors following participation in an intervention [18-21]. The acceptability of the workshop from the point of view of the health professionals was also assessed as part of the evaluation.

## Methods

### Workshop

The interactive workshop was developed based on the COM-B model [5] and provided information on the importance and use of behavior change techniques that could be used in everyday practice for opportunistic behavior change. There were five core objectives of the workshop: (1) to detail the role of health professionals in delivering optimal care; (2) to demonstrate opportunities to change behavior; (3) to describe the principles of behavior change; (4) to explain behavior change techniques; and (5) to determine the most appropriate behavior change techniques to use and when to use them.

The workshops were conducted in collaboration with the Pharmaceutical Society of Australia (PSA), Western Australia Division. Health professionals involved in the care of chronic disease patients, specifically those working with patients with heart failure, were invited to attend a professional development day organized by the PSA. The first component of the professional development day was run by a cardiologist and focused on educating the attendees on the medical aspects of heart failure. This was presented separately from our workshop and was not part of the intervention evaluation. The second and final component of the professional development day was the 2-hour behavior change workshop, which was facilitated by 3 of the authors. If attendees provided informed consent to participate in the research evaluation of the workshop, they completed a baseline survey before the workshop and a follow-up survey immediately after the workshop.

The workshop was presented in 3 overall sections (see Multimedia Appendix 1 for the workshop schedule). The first section introduced the COM-B model and how it can be applied in practice. An interactive case-study activity was then introduced. The case study provided an example of a woman who did not want to adhere to hypertension medication and showed how the COM-B model could be used to identify barriers and facilitators to behavior change. Each attendee spent approximately 10 minutes working on the case study, with the group reconvening after this time to debrief and share responses. The second section focused on why behavior is hard to change and highlighted some of the difficulties health professionals encounter with their own behavior and attitudes when trying to change the behavior of their patients. The third and final part of the workshop introduced behavior change techniques and provided a more in-depth overview of certain groupings of behavior change techniques that may be appropriate in practice. Seven individual groups of behavior change techniques were discussed: social support, self-monitoring of behavior, verbal persuasion for capability, focus on past success, planning, attitude change, and automaticity. These groups were selected using Cards for Change, a toolkit for behavior change developed by researchers at the University of Manchester and Manchester Metropolitan University [22]. These cards provide clear definitions of each technique and supporting example activities that can assist educators and trainers in teaching behavior change techniques.

After discussing the behavior change groupings, attendees were randomly placed into small groups and instructed to “choose two of the behavior change techniques that have just been introduced, explore when they might work best, and what the barriers to implementation may be.” After approximately 10 minutes, the attendees reconvened, and the workshop presenters facilitated debriefing and sharing of responses. The final activity was another group discussion activity, which asked attendees, in small groups, to complete the following task: “using the 12 behavior change techniques that have been introduced, explore what combinations might work best and in what circumstances.” A final debriefing was then facilitated by the workshop presenters.

### Study Design and Procedure

A prospective study design was used to implement and evaluate the behavior change workshop. A total of 10 workshops were conducted between September and November 2021, with 6 held in person in metropolitan Perth and 4 held online for those in regional areas. All workshop attendees were contacted by the PSA through its database of health professionals and were invited to attend the free professional development day. When signing up for the professional development day, all attendees provided basic demographic information to the PSA.

Prior to participating in the 2-hour workshop, all attendees were provided with a link to the survey, hosted on Qualtrics. The first page of the survey provided a participant information sheet and a consent form. Attendees that were interested in participating in the evaluation of the workshop were asked to provide informed consent for both time points after reading the participant information sheet. After providing consent, participants were asked to provide their first name and email address, so that their baseline and postworkshop surveys could be linked. No other demographic information was collected from participants. After this, participants completed questions related to their current knowledge of behavior change techniques, current use of behavior change techniques in practice, and the psychological variables. All attendees then participated in the workshop, and at the end they were provided another Qualtrics link to the follow-up survey. This survey was the same as the preworkshop survey, but had additional items related to the acceptability of the workshop.

### Ethical Considerations

Ethics approval was obtained from the Human Research Ethics Committee of the Curtin University, Australia (HRE2021-0567) prior to any data collection or facilitation of the workshops. Participants provided informed consent by marking a checkbox at the start of the preworkshop survey. Survey data were deidentified following the merging of participants’ responses from the pre- and postworkshop surveys. Participants were not provided compensation for their time, as the professional development workshop was provided to participants free of charge through the PSA.

### Measures

All psychosocial measures were based on the standardized procedures for measure development outlined by Ajzen [17]. This included defining the behavior and research population and formulating reflective and direct measures to address each of the main constructs of the Theory of Planned Behavior [23].

#### Perceived Knowledge

Participants’ perceived knowledge of behavior change techniques was assessed and measured both before and after the workshop using a single item: “On a scale of no understanding to perfect understanding, how would you rate your knowledge of behavior change techniques?” Participants rated their level of understanding on a 7-point Likert scale. Scores were summed to yield a total perceived knowledge score. Higher scores indicated greater perceived knowledge.

#### Attitudes

To measure attitudes toward behavior change techniques in practice, participants were provided with the single phrase “For me, changing my professional practice to reduce the effects of heart failure would be...” and were asked to complete this item for 2 attitudes, wisdom and usefulness, with responses on a sliding 7-point Likert scale, ranging from 1 (very wise/very useful) to (7 very unwise/very useless). Items were reverse scored and responses to the 2 items were averaged. Higher scores indicated positive attitudes toward behavior change techniques. The attitude measure demonstrated excellent internal consistency before and after the workshop (α=.98 and α=.95, respectively).

#### Social and Descriptive Norms

Two items were developed to assess social and descriptive norms related to changes in professional practice to incorporate behavior change techniques. These items were provided both pre- and postworkshop. Participants rated their agreement with each statement on a 7-point Likert scale, ranging from 1 (strongly disagree) to 7 (strongly agree). For descriptive norms, the item was “The people who are important to me think I should change my professional practice to reduce the effects of heart failure.” For subjective norms, the item was “People like me think I should change my professional practice to reduce the effects of heart failure.” Agreement with each norm was represented by a higher score.

#### Perceived Behavioral Control

Perceived behavioral control was measured using 1 item developed for this study. Participants rated the item “I am confident I can change my professional practice to reduce the effects of heart failure” on a 7-point Likert scale, ranging from 1 (strongly disagree) to 7 (strongly agree). Perceived behavioral control was measured both before and after the workshop. Higher perceived behavioral control to change behavior in professional practice was indicated by a higher score.

#### Intention

A single item was used to assess participants’ intention to use behavior change techniques over the next 4 weeks: “I intend to provide behavior change techniques to my heart failure patients over the next four weeks.” Participants rated how much they agreed with the statement on a 7-point Likert scale, ranging from 1 (strongly agree) to 7 (strongly disagree). Intention was measured both before and after the workshop. A higher score indicated greater intention to use behavior change techniques in practice.

#### Workshop Acceptability

Two items were used to assess the acceptability of the workshops. The items were constructed based on the feasibility and acceptability questionnaire developed by Kothe and Mullan [24]. The first set of items asked participants to rate their agreement with each of 7 statements on their feelings about whether the workshops were needed, useful, appropriate for the profession, applicable to their current practices, interesting, exciting, and worth their time. The statements were rated on a 5-point Likert scale, ranging from 1 (completely disagree) to 5 (completely agree). The internal consistency was good, with Cronbach α=.94. The second set of items asked participants to indicate how satisfied they were with the workshop on a 5-point Likert scale, ranging from 1 (completely dissatisfied) to 5 (completely satisfied). The statements were summed to create a total score. A higher score represented greater overall acceptance of the behavior change workshop. Higher scores on item 2 represented greater satisfaction with the workshop.

### Data Analysis

Pre- and postworkshop survey responses were matched using the participants’ email addresses, which were then removed and replaced with an anonymous participant ID. Data were screened for errors and missing values. Missing values were imputed using expectation maximization. Differences between pre- and postworkshop scores for knowledge, attitude, social and descriptive norms, perceived behavioral control, and intention were assessed using 2-tailed paired-samples *t* tests. We adjusted our α level for multiple comparisons with Bonferroni correction. Therefore, our α level for the paired-samples *t* tests was α=.008. Pearson correlations were also used to determine the association between pre- and postworkshop scores.

## Results

### Workshop Attendees

A total of 127 health professionals from Western Australia attended 1 of 10 workshops on the professional development day organized by the PSA. This sample of workshop attendees included general practitioners (n=4, 3.1%), nurses (n=16, 12.6%), and pharmacists (n=107, 84.3%). The majority of the workshop attendees were women (n=108, 85%), and just over half attended the in-person workshops (n=75, 59.1%).

### Survey Participants

Of the 127 attendees, 71 completed the preworkshop survey. Four participants did not provide consent, 10 participants did not complete any part of the survey, and 1 response was a duplicate. Removing these responses left data from 56 participants. The postworkshop survey was completed by 58 attendees; only 1 participant was removed due to not completing any items on the survey. The final data combined pre- and postworkshop survey responses. Seventy-two completed surveys were screened, and email addresses were matched. Of these completed surveys, 6 duplicates were removed. Twelve participants completed only the preworkshop survey and 13 completed only the postworkshop survey. A final sample of 41 participants who completed both the pre- and postworkshop surveys was used for the analyses.

The demographic information of the 41 survey participants was not collected. Although the PSA collected attendee demographics when they signed up for the professional development day, due to the anonymization of the survey responses, the demographics of the survey participants could not be linked to the PSA data. However, given the large proportion of workshop attendees who were pharmacists and the large proportion of women, it is likely that most survey participants were pharmacists and that most were women.

### Correlations Between Pre- and Postworkshop Psychosocial Variables

Table 1 shows the Pearson correlations between pre- and postworkshop psychosocial variables.

**Table 1:** Pearson correlations for pre- and postworkshop psychosocial variables.

Variable				1		2		3		4		5		6		7		8		9		10		11		12		13		14		15		16		
**Pre workshop**																																				
	**1 Knowledge**																																			
		*r*	1		–0.10		–0.13		–0.11		0.15		0.02		0.23		0.13		0.04		–0.04		–0.04		–0.04		0.25		0.09		0.28		0.33			
		*P* value	—^a^		.60		.48		.54		.36		.90		.15		.41		.82		.80		.80		.80		.11		.51		.70		.05			
	**2 Attitude (wise/unwise)**																																			
		*r*	–0.10		1		0.98		0.99		–0.16		–0.19		0.25		0.19		0.17		0.59		0.56		0.59		–0.07		0.04		0.10		0.04			
		*P* value	.60		—		<.001		<.001		.30		.23		.12		.23		.29		<.001		<.001		<.001		.66		.79		.53		.79			
	**3 Attitude (useful/useless)**																																			
		*r*	–0.13		0.98		1		0.99		–0.20		–0.22		0.20		0.16		0.18		0.53		0.51		0.53		–0.11		0.02		0.09		0.06			
		*P* value	.48		<.001		—		<.001		.21		.17		.21		.33		.26		<.001		<.001		<.001		.50		.90		.58		.70			
	**4 Attitude average**																																			
		*r*	–0.11		0.99		0.99		1		–0.18		–0.21		0.23		0.17		0.18		0.56		0.54		0.56		–0.09		0.03		0.10		0.05			
		*P* value	.54		<.001		<.001		—		.25		.20		.16		.28		.28		<.001		<.001		<.001		.57		.84		.53		.75			
	**5 Descriptive norms**																																			
		*r*	0.15		–0.16		–0.20		–0.18		1		0.65		0.29		0.16		–0.13		–0.19		–0.27		–0.24		0.70		0.47		0.10		0.17			
		*P* value	.36		.30		.21		.25		—		<.001		.07		.32		.43		.23		.08		.13		<.001		.002		.53		.28			
	**6 Subjective norms**																																			
		*r*	0.02		–0.19		–0.22		–0.21		0.65		1		–0.02		0.03		0.08		–0.22		–0.24		–0.23		0.69		0.51		0.06		0.13			
		*P* value	.90		.23		.17		.20		<.001		—		.88		.85		.63		.18		.13		.14		<.001		<.001		.69		.43			
	**7 Perceived behavioral control**																																			
		*r*	0.23		0.25		0.20		0.23		0.29		–0.02		1		0.78		0.17		0.15		0.10		0.13		0.07		0.05		0.54		0.50			
		*P* value	.15		.12		.21		.16		.07		.88		—		<.001		.28		.34		.54		.43		.68		.76		<.001		<.001			
	**8 Intention**																																			
		*r*	0.13		0.19		0.16		0.17		0.16		0.03		0.78		1		0.38		0.13		0.14		0.14		0.04		–0.02		0.52		0.54			
		*P* value	.41		.23		.33		.28		.32		.85		<.001		—		.02		.43		.39		.40		.82		.93		<.001		<.001			
**Postworkshop**																																				
	**9 Knowledge**																																			
		*r*	0.04		0.17		0.18		0.18		–0.13		0.08		0.17		0.38		1		0		0.04		0.02		–0.04		–0.12		0.31		0.31			
		*P* value	.82		.29		.26		.28		.43		.63		.28		.02		—		1		.83		.91		.81		.44		.052		.053			
	**10 Attitude (wise/unwise)**																																			
		*r*	–0.04		0.59		0.53		0.56		–0.19		–0.22		0.15		0.13		0		1		0.92		0.98		–0.04		0.09		0.32		0.15			
		*P* value	.80		<.001		<.001		<.001		.23		.18		.34		.43		1		—		<.001		<.001		.81		.58		.05		.35			
	**11 Attitude (useful/useless)**																																			
		*r*	–0.04		0.56		0.51		0.54		–0.27		–0.24		0.10		0.14		0.04		0.92		1		0.98		–0.08		0.05		0.27		0.13			
		*P* value	.80		<.001		<.001		<.001		.08		.13		.54		.39		.83		<.001		—		<.001		.61		.74		.08		.43			
	**12 Attitude average**																																			
		*r*	–0.04		0.59		0.53		0.56		–0.24		–0.23		0.13		0.14		0.02		0.98		0.98		1		–0.06		0.07		0.30		0.14			
		*P* value	.80		<.001		<.001		<.001		.13		.14		.43		.40		.91		<.001		<.001		—		.70		.66		.06		.38			
	**13 Descriptive norms**																																			
		*r*	0.25		–0.07		–0.11		–0.09		0.70		0.69		0.07		0.04		–0.04		–0.04		–0.08		–0.06		1		0.69		0.21		0.26			
		*P* value	.11		.66		.50		.57		<.001		<.001		.68		.82		.81		.81		.61		.70		—		<.001		.18		.10			
	**14 Subjective norms**																																			
		*r*	0.09		0.04		0.02		0.03		0.47		0.51		0.05		–0.02		–0.12		0.09		0.05		0.07		0.69		1		0.15		0.26			
		*P* value	.51		.79		.90		.84		.002		<.001		.76		.93		.44		.58		.74		.66		<.001		—		.37		.10			
	**15 Perceived behavioral control**																																			
		*r*	0.28		0.10		0.09		0.10		0.10		0.06		0.54		0.52		0.31		0.32		0.27		0.30		0.21		0.15		1		0.84			
		*P* value	.70		.53		.58		.53		.53		.69		<.001				.052		.05		.08		.06		.18		.37		—		<.001			
	**16 Intention**																																			
		*r*	0.33		0.04		0.06		0.05		0.17		0.13		0.50		0.54		0.31		0.15		0.13		0.14		0.26		0.26		0.84		1			
		*P* value	.05		.79		.70		.75		.28		.43		<.001		<.001		.053		.35		.43		.38		.10		.10		<.001		—			

^a^Not applicable.

### Knowledge of Behavior Change Techniques

A paired-samples test was used to evaluate the impact of the behavior change workshop on participants’ perceived knowledge of behavior change techniques before (mean score 3.73, SD 1.55) and after (mean score 5.20, SD 0.93) the workshop. There was a difference in mean knowledge scores of –1.46 (95% CI –2.02 to –0.90). This difference was significant (t_40_=–5.27, *P*<.001) and had a large effect size (Cohen *d*=1.78).

### Attitude

A paired-samples *t* test revealed no significant increase in attitude scores from before to after the workshop (t_40_=0.78, *P*=.44). There was a small difference in scores before (mean score 6.16, SD 1.27) and after (mean score 5.99, SD 1.63) the workshop, with a change in mean score of 0.17 (95% CI –0.27 to 0.61), but this was not a significant change.

### Social Norms

A paired-samples *t* test comparing social norms before (mean score 4.78, SD 1.29) and after (mean score 5.46, SD 1.23) the workshop revealed a change in mean score of –0.68 (95% CI –1.08 to –0.29). This change was significant (t_40_=–3.49, *P*=.001) and had a large effect size (Cohen *d*=1.25).

### Descriptive Norms

A paired-samples *t* test was conducted and showed a significant change in descriptive norms from before to after the workshop (t_40_=–3.65, *P*<.001). The mean score for descriptive norms changed by –0.61 (95% CI –0.95 to –0.27) from before (mean score 4.49, SD 1.47) to after (mean score 5.10, SD 1.22) the workshop. The effect size for this test was large (Cohen *d*=1.07).

### Perceived Behavioral Control

A paired-samples *t* test revealed a significant change in perceived behavioral control scores from before (mean score 5.33, SD 1.15) to after (mean score 5.83, SD 0.74) the workshop (t_40_=–3.30, *P*=.002). This represented a change in mean score of –0.50 (95% CI –0.81 to –0.20). There was a large effect size (Cohen *d*=.98).

### Intention

A paired-samples *t* test was conducted to examine if there was a significant change in intention from before (mean score 5.30, SD 0.98) to after (mean score 5.75, SD 0.84) the workshop. There was a significant difference in intention (t_36_=–3.32, *P*=.002), with a mean score increase of –0.46 (95% CI –0.74 to –0.18) and a large effect size (Cohen *d*=0.88).

### Workshop Acceptability

Following the workshop, 78% (32/41) of participants agreed or strongly agreed that the workshop was needed, and the remainder neither agreed nor disagreed. No participant felt that the workshop was not needed. Participants also felt that the workshop was useful, with 85% (35/41) of participants agreeing, while the rest neither agreed nor disagreed. There were no participants who felt that the workshop was not useful. The majority of the participants felt that the training was appropriate for their profession, with 90% (37/41) of participants agreeing, while 10% (4/41) were undecided.

Workshop participants completely agreed or agreed that the workshop was applicable to their current practices (41/47, 90%), while 10% (4/41) were neutral. Most participants indicated that the workshop was interesting (35/41, 85% agreed or completely agreed), while 14% (5/41) were neutral and 3% (1/41) participants completely disagreed. Most participants agreed or completely agreed (33/41, 80%) that the workshop was worth their time, while only a small proportion of participants completely disagreed or disagreed (2/41, 5%) and only 15% (6/41) were neutral. Overall, participants demonstrated a high acceptance of the workshop (mean score 28.84, SD 4.44). Further, most participants were satisfied or completely satisfied (36/41, 88%) with the 2-hour workshop.

## Discussion

### Principal Results

The 2-hour behavior change workshop was effective in changing knowledge, social and descriptive norms, perceived behavioral control, and intention, but was not effective at changing attitudes. Further, health professionals found that the workshop was acceptable, and they were satisfied overall with the content and delivery of the behavior change workshops.

Knowledge of behavior change techniques can lead to changes in important psychosocial predictors of behavior. While knowledge alone is generally insufficient to change behavior, behavior change is more likely to occur with improvements in knowledge [25]. Thus, other skills and techniques are required to ensure health professionals have the capability, opportunity, and motivation to change behavior. As suggested by Ruppar et al [26], no intervention focused on changing behavior should focus solely on patient education. Rather, patient education works best when it is combined with more active behavioral approaches. Thus, in the context of training health professionals, increasing their knowledge of behavior change techniques and providing them opportunities to discuss case studies and real-life applications of the techniques increases the likelihood of sustained changes in behavior. Increasing the participants’ perceived knowledge of behavior change techniques alone does not ensure they will apply these techniques; previous research focused on improving health care professionals’ knowledge and practices regarding adverse drug reactions showed that providing an intervention targeting both knowledge and other psychosocial variables had positive effects on professional practice at the end of 12 months [27]. However, the long-term effectiveness of such interventions and the training of health professionals beyond a 12-month follow-up is uncertain; future longitudinal research is required [13,27].

Both social and descriptive norms also significantly improved after the workshop. Improvement in norms, both descriptive (referring to people important to them) and social (referring to people like them), is theorized to increase health care professionals’ intention to complete behaviors, in this case, the use of behavior change techniques with their patients. The workshops increased norms with a large effect size. Norms may have been improved by a multitude of workshop components, in particular the increase in knowledge of behavior change techniques. Across various domains, educational interventions that increase knowledge often result in improved ratings for subjective norms [28-30]. In the workshops, an increase in knowledge of behavior change techniques, including their evidence base and why and how they are used, may have increased health care professionals’ perception of whether others (descriptive norms) and people similar to them (social norms) believe they should change their professional behaviors to ensure optimal care.

Participants’ attitudes toward changing their professional practice to provide optimal care did not change significantly. Preworkshop attitudes were high, with a mean score of 6.16 on a scale from 1 to 7. Therefore, it is likely that the lack of significant improvement in attitudes following the workshop was due to a ceiling effect, in that improvements to attitudes were unlikely due to high preintervention levels. This has previously been seen in studies of the Theory of Planned Behavior–based interventions [18,31] and suggests that improvements to other components of the modes (ie, norms and perceived behavioral control), rather than attitudes, are required to improve intention and thereby behavior.

The 2-hour workshop intervention also resulted in large improvements to participants’ ratings of perceived behavioral control. The Theory of Planned Behavior posits that perceived behavioral control not only influences intention to perform a behavior, but also directly predicts behavior [17]. Therefore, the demonstrated postworkshop improvements in perceived behavioral control show particular promise for not only improving participants’ intention to use behavior change techniques, but also to facilitate the actual use of behavior change techniques with their patients.

Pre- and postworkshop comparisons revealed a significant improvement in the participants’ intention to use behavior change techniques with their patients. This is unsurprising, as the Theory of Planned Behavior posits that changes in attitudes, norms, and perceived behavioral control will result in increased intention, and within our sample, norms (both descriptive and social) and perceived behavioral control significantly improved, with large effect sizes. Further, the Theory of Planned Behavior theorizes that intention directly predicts behavior [17]. Therefore, in the context of the intervention, improvements in the participants’ intention should lead to the use of behavior change techniques with their patients. Indeed, a great deal of previous research has demonstrated that intention is a statistically significant predictor of behavior [18,32]. However, it is also important to note that the body of literature points toward an intention-behavior gap, as intention often only accounts for a limited amount of variance in behavior [33,34]. Therefore, although our intervention shows promise in facilitating the participants’ use of behavior change techniques with their patients, future interventions might also incorporate techniques that lead to habitual use of behavior change techniques with patients to ensure more consistent use of behavior change techniques in the health domain [35-38]. However, this was beyond the scope of this intervention.

### Limitations and Directions for Future Research

A key limitation of the current study was the high attrition from before to after the workshop, with the final comparison sample including only 58% (74/127) of those that completed the preworkshop survey. A related limitation is that we had an inadequate sample size to use analyses, such as structural equation modeling, that are part of the Theory of Planned Behavior, thus limiting our analysis to pre- and postworkshop comparisons. However, it is also important to note that assessing the relationships between the components of the Theory of Planned Behavior was not an aim of this study. Future research should seek to replicate our study with larger sample sizes that allow for the assessment of the fit of our theoretical model, using analyses such as structural equation modeling and confirmatory factor analysis [18,39-42].

A further limitation of the study design was that some items on the pre- and postworkshop surveys only contained a single item. This was done to facilitate practical assessment and to limit the burden on participants, thereby increasing validity [43]. Further, this approach has been shown to be valid in previous work [44]. However, future research should further validate our findings by using measures with multiple items for each domain. In addition, due to not having collected any demographic variables of the participants, we were unable to evaluate if there were any differences based on participant demographics (eg, pharmacist vs nurse) or the workshop delivery mode (eg, online vs face-to-face). This limited our understanding of how and for whom the intervention was most effective. Future research should consider evaluating any demographic differences between workshop participants to inform more targeted future interventions.

An additional limitation to this study was that there was no subsequent follow-up. This makes it difficult to know if the changes in psychosocial factors were maintained over time or if there is a need to engage health professionals in regular training in behavior change to sustain long-term changes in these psychosocial factors. Further, we could not assess the frequency of the participants’ application of behavior change techniques over time. Future research should explore the lasting impacts of the intervention and include a measure of health professionals’ behavior to determine the translation of the improvements to knowledge, subjective norms, descriptive norms, perceived behavioral control, and intention in the health professionals’ practices over time.

Lastly, the participants demonstrated high attitude scores in the preworkshop survey, which suggests that there may have been some self-selection bias. The design of the study did not assess if participants had prior training, knowledge, or interest in behavior change, which may have biased the results. Self-selection bias has been noted as a limitation in other health behavior interventions [45,46] and future research should consider strategies to mitigate self-selection bias by including control groups (eg, wait-list control groups) to improve the internal validity of the study design.

This study did, however, use a theoretically informed and evidence-based workshop design incorporating both active and passive components. The participants came from a range of disciplines and our results demonstrate the effectiveness and acceptability of our intervention across different health professions.

### Conclusion

Ultimately, the 2-hour, theoretically informed, evidence-based workshop designed to facilitate the use of behavior change techniques by health professionals was shown to be largely effective. The workshops resulted in a significant increase in knowledge, descriptive norms, subjective norms, perceived behavioral control, and intention. The intervention was also shown to be highly acceptable for many participants, who deemed the intervention needed, useful, appropriate, and applicable, as well as interesting and worth their time. Future research should explore the lasting impacts of the workshop on health professionals’ practices, as well as how changes in their practices may impact their patients in clinical and community settings.

## Appendix

Multimedia Appendix 1 Workshop Schedule.

## Abbreviations

COM-B: Capability, Opportunity, Motivation, and Behavior
PSA: Pharmaceutical Society of Australia

## Data Availability

The data sets generated during and analyzed during the current study are not publicly available due to privacy and ethical restrictions but are available from the corresponding author on reasonable request.
