# Supplementary material for: Behavior Change Training for Health Professionals: Evaluation of a 2-Hour Workshop
Source: JMIR Form Res. 2022 Nov 18;6(11):e42010. doi: 10.2196/42010 (PMC9719063; doi:10.2196/42010)
Supplement: Multimedia Appendix 1 [file formative_v6i11e42010_app1.pdf]

Multimedia Appendix 1: Workshop Schedule

Session	Duration	Topics
Introduction	10 minutes	A short introduction was provided to introduce the presenters and the plan for the workshop session. An outline of the following learning objectives was provided.<br>**By the end of the session participants should be able to:**<br>• Recognise how to optimise patient care within your practice setting through the implementation of heart failure guidelines.<br>• Outline the role of the health professional in delivering optimal care<br>• Demonstrate opportunities to change behaviour<br>• Describe principles of behaviour change<br>• Explain behaviour change techniques<br>• Determine the most appropriate behaviour change techniques to use and when
COM-B model	20 minutes	In section one of the workshop, the Capability, Opportunity, Motivation, Behaviour (COM-B) model was introduced to participants. In this section of the workshop, the capability, opportunity and motivation for behaviour change of patients was explored, with information provided on what strategies can be used to change these components (e.g., training, clear messaging, modelling, rewards).
Case study	15 minutes	This section of the workshop provided participants with a case scenario of a patient who needs to change their behaviour to reduce their risk of heart failure, utilising a multidisciplinary team approach. This case scenario was provided in terms of how the COM-B model might be used in this scenario.
Debrief	5 minutes	A debrief following the case study was provided allowing for feedback and discussion within the group of participants.
Behaviour change techniques A	20 minutes	Section two of the workshop provided participants with knowledge surrounding why behaviour is hard to change, highlighting areas of environmental context and resources, beliefs about consequences, professional role and beliefs about capabilities. Information on simple versus complex behaviours was also provided. In this section, participants explored what health professionals can do to make it easier for patients to change their behaviour (e.g., understand lived experiences, competing goals, variations in motivation). An introduction to behaviour change techniques was also included in this section.
Behaviour change techniques B	15 minutes	Section three provided participants with an in-depth overview of seven individual groups of behaviour change techniques including social support, self-monitoring of behaviour, verbal persuasion about capability, focus on past success, planning, attitude change, and automaticity. In this section participants were provided information on which techniques work best and when, what are the barriers and benefits to their use and how can these techniques link to a patient's Capability, Motivation and Opportunity for Behaviour change.
Group activity 2	15 minutes	The final group activity invited participants to choose two of the behaviour change techniques that were introduced, and to explore when they might work best and what the barriers to implementation might be.
Debrief	5 minutes	A debrief following the group activity was provided allowing for feedback and further discussion among participants.
Q & A<br>Summary	15 minutes	At the end of the workshop, participants were provided with the key take home messages and some resources to support their understanding of behaviour change and behaviour change techniques.
